# Traffic surveillance video-based a unified road accident detection, classification, and vehicle tracking framework using an optimized spatio-temporal attention mechanism

**DOI:** 10.1371/journal.pone.0357111

**Published:** 2026-10-15

**Authors:** Chokkakula Devi, Gowri S.

**Affiliations:** 1 Department of Computer Science and Engineering, Sathyabama Institute of Science and Technology (Deemed to be University), Chennai, Tamil Nadu, India; 2 School of Computing, Sathyabama Institute of Science and Technology (Deemed to be University), Chennai, Tamil Nadu, India; Mae Fah Luang University, THAILAND

## Abstract

In recent days, vehicular accident detection has materialized because of the unique and intriguing application potential in the evolution of Intelligent Transportation Systems (ITS). To monitor and respond to the event of a fatal accident, real-world traffic surveillance footage requires continuous analysis. In this research, the road accident from the traffic surveillance video is detected, classified, and vehicle tracked using an efficient and innovative model. The surveillance videos are gathered from the CCTV Footage dataset, which is a publicly available dataset. The gathered videos are directly applied to the Spatio-Temporal Attention-based Adaptive Yolov9 with SE-InceptionNet and Deep SORT (AYSIDSORT) model for detecting, classifying and tracking traffic accidents very effectively. The developed network allows accurate detection and tracking of vehicles in real-time, which ensures the continuous traffic flow without losing moving objects. The variables of the proposed AYSIDSORT model are tuned by the developed Generation Probability-based Equilibrium Optimizer (GP-EO) to improve the detection and classification performance. The exact location of the accident is sent to the appropriate entities using an Internet of Things (IoT) platform to provide a timely rescue for the affected individual. Here, the proposed framework acts as a computer vision-based accident detection and tracking framework with potential IoT integration to find the exact location of the accident, which helps to provide a timely rescue for the affected individual. The effectiveness of the offered approach is validated with various baseline detection models. The accuracy of the suggested AYSIDSORT reaches to 95% in the 100^th^ epoch, whereas there is a slight variation in the accuracy values when the batch size is varied from 4 to 128. The outcomes show that the designed framework is better than existing methods.

## 1. Introduction

Road traffic management members and authorities are under stress to handle problems, such as traffic accidents, as a consequence of the growing number of vehicles on the road, so prompt surveillance is necessary to reduce the probability of fatalities and property damage [1]. One of the primary issues with traffic management is vehicle accidents on major roads and highways. When accidents happen, it is critical to report them right away. When traffic accidents are automatically detected, traffic can return to normal [2]. Furthermore, CCTV cameras continuously stream their footage, and manual surveillance is prone to mistakes [3]. For sustainable transportation and improved traffic flow management, the development of an automated model for identifying anomalous occurrences, such as accidents, is necessary [4].

Each year, the number of daily traffic accidents rises, leading to a considerable number of fatalities and disability [5]. Speeding, careless driving, the presence of stray animals on the road, driver fatigue, and insufficient infrastructure are the primary causes of traffic accidents [6]. The majority of fatalities and disabilities are caused by emergency medical assistance response [7]. In recent years, considerable resources have been devoted to the purpose of delivering effective and timely rescue operations [8]. One tool that has been used to achieve this is the Internet of Things (IoT) [9]. The IoT has recently been used to improve passenger and driver safety [10]. IoT systems for detecting, localizing, reporting, modelling, and analyzing traffic accidents have been presented in some research studies [11]. The primary disadvantages of the current accident detection systems are their exorbitant price and exclusivity for luxury automobiles [12]. The majority of these systems rely on smartphones, which can malfunction and vary the results if they are thrown outside the car [13]. Commonly, IoT provides improved device, system, and service connections spanning some protocols, domains, and applications that extend beyond Machine-to-Machine (M2M) interactions.

All of the major cities in the world have already deployed cameras for traffic monitoring purposes due to various security concerns [14]. Although using the current networks of surveillance cameras will be a practical solution, the majority of these systems rely on human observation [15]. It is nearly impossible for human observers to identify anomalous activities in various real-time camera scenarios [16]. As a result, automated accident detection solutions are necessary [17]. It is very difficult to develop a vision-based algorithm for this task. Techniques based on computer vision are extensively used for accident detection and intelligent traffic surveillance [18]. However, different lighting and traffic situations have an impact on computer vision-based systems [19,20]. The exceptional performance of machine learning techniques in representing visual data is responsible for immense success [21,22]. By viewing traffic accidents as a unique example of anomalous activity [23], a machine learning method for detecting and localizing traffic accidents has been proposed. Travel safety would be made possible by detecting and localizing traffic accidents, which would also help rescue teams respond more quickly.

The contributions of the proposed work are discussed below:

- To develop an IoT-driven road accident detection and tracking approach for determining road accidents, for providing timely medical aid and also for improving the life span of humans. Hospitals can provide immediate medical aid by alerting the nearby medical centre through the recommended accident detection and tracking approach. The casualties occurred because of the improper and timely medical assistance being prevented by using the recommended model. With the utilization of the proposed model, the authorities can implement precise preventive measures for reducing the possibility of accidents.
- To present the AYSIDSORT model for detecting and tracking road accidents to prevent the loss of human life due to delays in ambulance arrival at the accident site. The suggested AYSIDSORT model is developed with the combination of spatio-temporal attention, Yolov9, SE-InceptionNet and Deep SORT approaches for accurately detecting and tracking road accidents in real-time. The developed AYSIDSORT model facilitates the effective road accident detection while reducing both the missed detection and false alarms. The developed approach continuously processes the video frames from traffic cameras using the YOLOv9 model for detection, the attention mechanism focuses on the relevant parts of the images, and Deep SORT for tracking the vehicles associated with the accidents.
- To suggest GP-EO for optimizing the parameters of the Yolov9, SE-InceptionNet and DeepSORT models for maximizing the traffic accident detection and classification performance. Here, the GP-EO balances the convergence and efficiency by precisely searching the high-dimensional search space, which helps to select the appropriate hyperparameters and to maximize the accuracy in the classification of road accidents. Moreover, the efficacy of the road accident detection and tracking is improved by the GP-EO as it converges towards the direction of the global optima by upgrading the solution searching performance of conventional EO.

The organization of the paper is given as below: The analysis of the traffic accident prediction and tracking is given in Section 2. The detailed preview of the implemented road accident detection and tracking method in an IoT environment is given in Section 3. The deep learning architectures used for traffic accident detection are given in Section 4. The detailed workflow of the IoT-oriented road accident detection and tracking framework is discussed in Section 5. The simulation setting and the comparison of the existing models are described in Section 6. The concluding remarks are finally discussed in Section 7.

## 2. Literature survey

### 2.1. Related works

In 2022, Pawar and Attara [24] designed a Sequence-to-Sequence Long Short-Term Memory Autoencoder (seq2seq-LSTM-AE) and a spatio-temporal autoencoder to model the video’s temporal and spatial representations. The datasets were used to test the model, and noteworthy qualitative and quantitative results were obtained.

In 2022, Sui *et al.* [25] designed an automatic approach for identifying and evaluating traffic accidents. Next, to locate the wrecked cars in the proper area, the YOLOV3 model was used. The corresponding trajectories were obtained by applying a hierarchical clustering approach before the collision.

In 2024, Kheder *et al.* [26] introduced an IoT-aided deep learning model for performing real-time traffic accident detection. The traffic sign detection was performed through LeNet-5 and a transfer learning-aided Inception-V3 approach. The optimal distance was determined to reduce road accidents. The developed LeNet-5 achieved higher accuracy rates over conventional traffic monitoring approaches. The simulation outcomes illustrated the effective traffic accident detection performance of the presented method.

In 2024, Adewopo *et al.* [27] suggested a lightweight deep learning approach to explicitly perform accident detection in vehicle monitoring systems. Empirical analysis on the developed lightweight model verified the impressive performance of the designed approach. The experimental results verified the effectiveness in a limited number of datasets, traffic scenarios, and road structures. The experimental results showed a more impressive performance of the suggested vision-based accident detection system in smart urban infrastructures. The developed automatic accident detection and classification effectively reported the type of accidents.

In 2022, Bakheet and Al-Hamadi [28] introduced a novel vision-based framework for the prediction and classification of vehicle accidents in real-time. The suggested approach started by automatically detecting moving objects and then tracking those objects dynamically using clustering. The temporal templates of moving objects were then used to extract a large collection of local features. The extracted features were then used to train a computationally efficient Deep Neural Network (DNN) model to identify anomalous vehicle behaviour patterns.

In 2022, Tamagusko *et al.* [29] suggested a model to identify possible accidents. After testing some Convolutional Neural Network (CNN) models, the transfer learning was the most effective strategy for traffic accident detection.

In 2020, Ghahremannezhad [30] introduced a new method for real-time traffic accident classification. The traffic direction was estimated in this research. Third, the decision-making system based on first-order logic was used to identify traffic accidents. Results from experiments using actual traffic video data demonstrated that the suggested approach was feasible.

In 2022, Balfaqih *et al.* [31] designed an IoT-based framework for classifying and detecting accidents. Based on the severity degree, the system identified and categorized the car crashes and sent the necessary accident data to emergency services providers.

### 2.2. Problem statement

The major causes of death are because of the unmonitored traffic on highways. The data on the occurrence of the accident is not communicated properly to the respective authorities, leading to an increased number of accidental deaths [32]. The continuous monitoring of the surveillance video for the detection of any abnormalities is a tiring process. Also, automatic accident detection from the surveillance video is affected by the presence of obstacles, illumination, and various other external factors [33]. Thus, a machine learning-based accident detection system is developed. The features and disadvantages of the existing accident detection and localization are illustrated in Table 1.

**Table 1:** Advantages and shortcomings of the baseline road accident detection and tracking models.

Author [citation]	Framework	Advantages	Shortcomings
Pawar and Attara [10]	Seq2Seq-LSTM-AE	• This method can be trained in normal traffic events and yet can effectively detect the occurrence of road accidents more accurately.	• This method takes more time to process the surveillance video.
Sui *et al.* [11]	YOLOV3	• This method is able to estimate the trajectories in which the vehicles travel after the occurrence of an accident.	• The detection of smaller dimensional objects is not possible by this method.
Singh and Mohan [13]	SVM	• This method is flexible, generalized, and can be used to detect accidents in any scenario. • It does not face any overfitting issues. • The effective localization of the accidents is also possible by this method.	• The detection rate of this method is affected by illumination change, changes in traffic flow, and the presence of obstacles.
Kumar *et al.* [15]	NB	• The type of accident can be determined by utilizing this method. • Retrofitting is possible in this approach based on the application. • This method is inexpensive and is able to send emergency messages within a short period.	• This approach is inconvenient to use as it uses two separate Android applications for execution.
Bakheet and Al-Hamadi [16]	Fuzzy vision-based DNN	• The detection performance offered by this method is better. • This method has a lower false positive rate even when predicting the accidents in real-time scenarios.	• This method is not very robust when provided with varying severity-based video inputs.
Tamagusko *et al.* [17]	CNN	• This method can effectively detect the probability of the accident event from both synthetic and real images.	• This method is not generalized to any work under any condition. • This method requires human intervention to provide accurate results.
Ghahremannezhad [19]	GFM, MOG, and NB	• This method can predict the accident events by estimating the traffic and the direction of movement of the traffic.	• This method is affected by zero-frequency issues.
Balfaqih *et al.* [20]	CART	• This method is able to predict the accident and classify the severity of the accident with higher precision.	• This method is not practically possible to implement.

- The processing of real-time surveillance video by conventional machine learning approaches is a slower and more tedious process. Hence, this research adopted the parallel cascaded machine learning model that highly reduces the total time of the road accident detection to save the lives of humans [34].
- The utilization of the entire video features makes the detection process inaccurate and more time-consuming. So, this research work adopted optimal weighted feature fusion that fuses necessary features obtained from the different models to make accurate results in the road accident detection and tracking [35].
- Most of the utilized features for the detection process do not show invariance to photometric and geometric changes in the video, which also leads to inaccurate detection outcomes. Here, different types of features are extracted from the entire video frames, which help to provide accurate and reliable results in the road accident detection and tracking process [36].

## 3. Detailed preview of the implemented road accident detection and tracking framework in an IoT environment

### 3.1. System model of IoT-based transportation systems

The objects are equipped with an actuator and sensor in the IoT paradigm, and this sensor is used for the main purpose of communicating with the processor and the actuator. The interconnection of the data is facilitated by the IoT, which helps to make the device much smarter. Each corner of the world is connected by the IoT, and it is more responsible for making communications between the digital world and the real world. A plethora of actuators and sensors are used for maintaining the communication between the digital world and the physical world. The processor, actuator and sensor are the major components of the IoT. The communication with the physical environment is possible through the sensors within the network. The processor can manage the data gathered from the sensor and store it securely. Depending on the value from the sensor, the required action is performed by the actuator. At that remote server, the data storage and processing are executed. The wireless communication is made between the IoT devices. The core infrastructure of the IoT is formed by the sensor, compute server and actuator. The geometric coordinates are regularly updated by the E-tracker device with inbuilt GPS, and it is serially connected with the Node MCU. The GPS coordinates are updated and transmitted by the E tracker towards the node MCU. Then, the IoT database gets the updated vehicle location, which is taken from the GPS coordinates. At each second, the GPS coordinates are obtained through the Think Speck cloud. The geographical location and Google Maps features are accessed by the Google Maps API. From the Think Speak Cloud, the GPS coordinates are read out through the Google Map API and visualize the location of the vehicle on the mobile app of the server. The system model of the ITS based on IoT is given in Fig 1.

**Fig 1:**
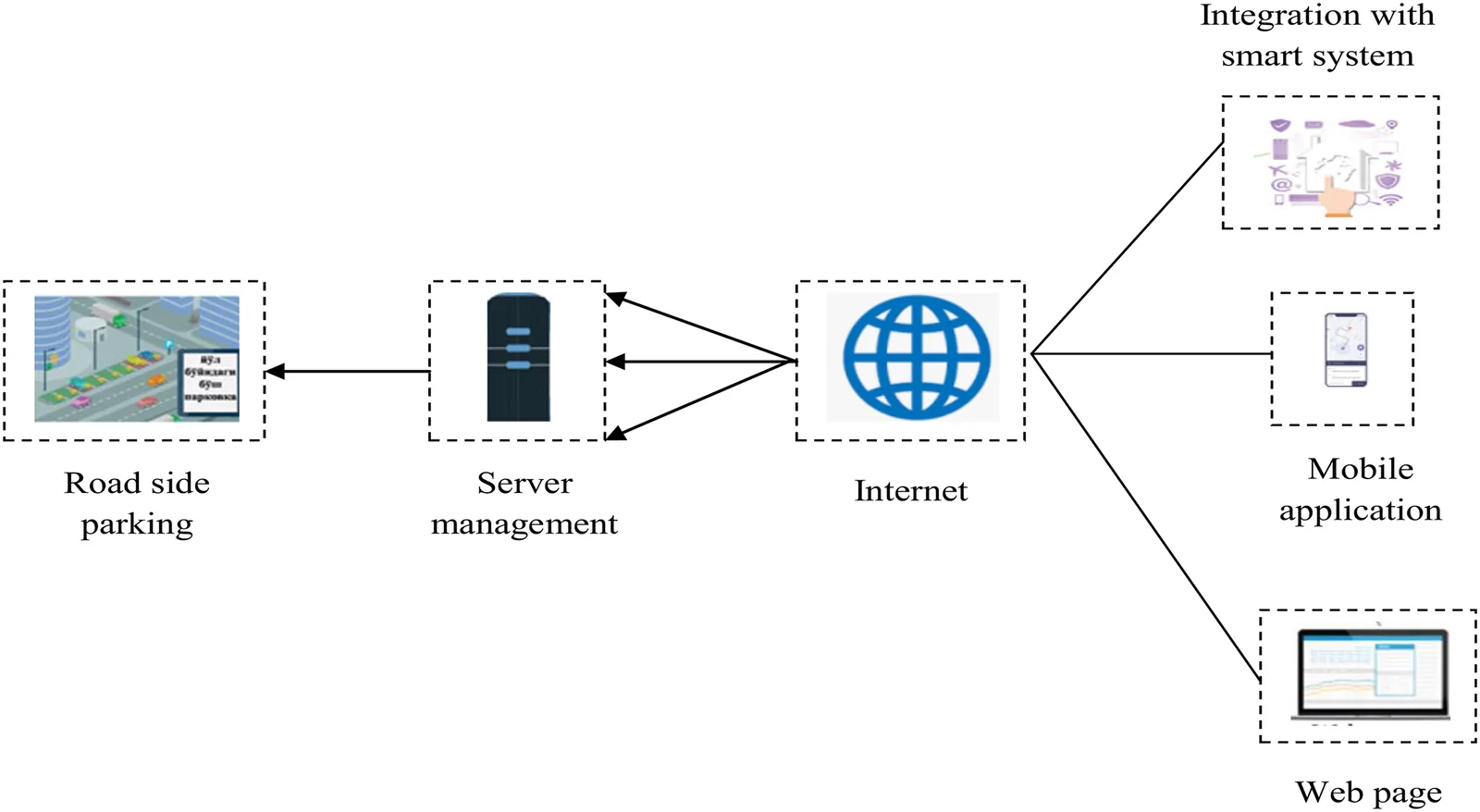
System model of the IoT-based ITS.

### 3.2. Architecture of the Implemented Approach

One of the emerging topics in the traffic monitoring system is called road accident tracking. The surveillance cameras are connected with the traffic management system in recent days; thus, the automated road accident detection is viably performed using the computer vision model. Over the last few decades, some breakthroughs in the platform of road accident detection and tracking have been proposed. The efficiency and the precision of road accident detection have been improved by numerous researchers. But the existing methods haven’t achieved higher accuracy in the detection of traffic accidents. In some cases, the previous models necessitate manually labelled images for the recognition of road accidents and the computational power of those models is very high. Some models require a lengthy training time to execute the road accident detection. So, this research presented a reliable and automated approach for the recognition of road accidents from the road surveillance footage. The graphical illustration of the implemented road accident detection and tracking model is given in Fig 2.

**Fig 2:**
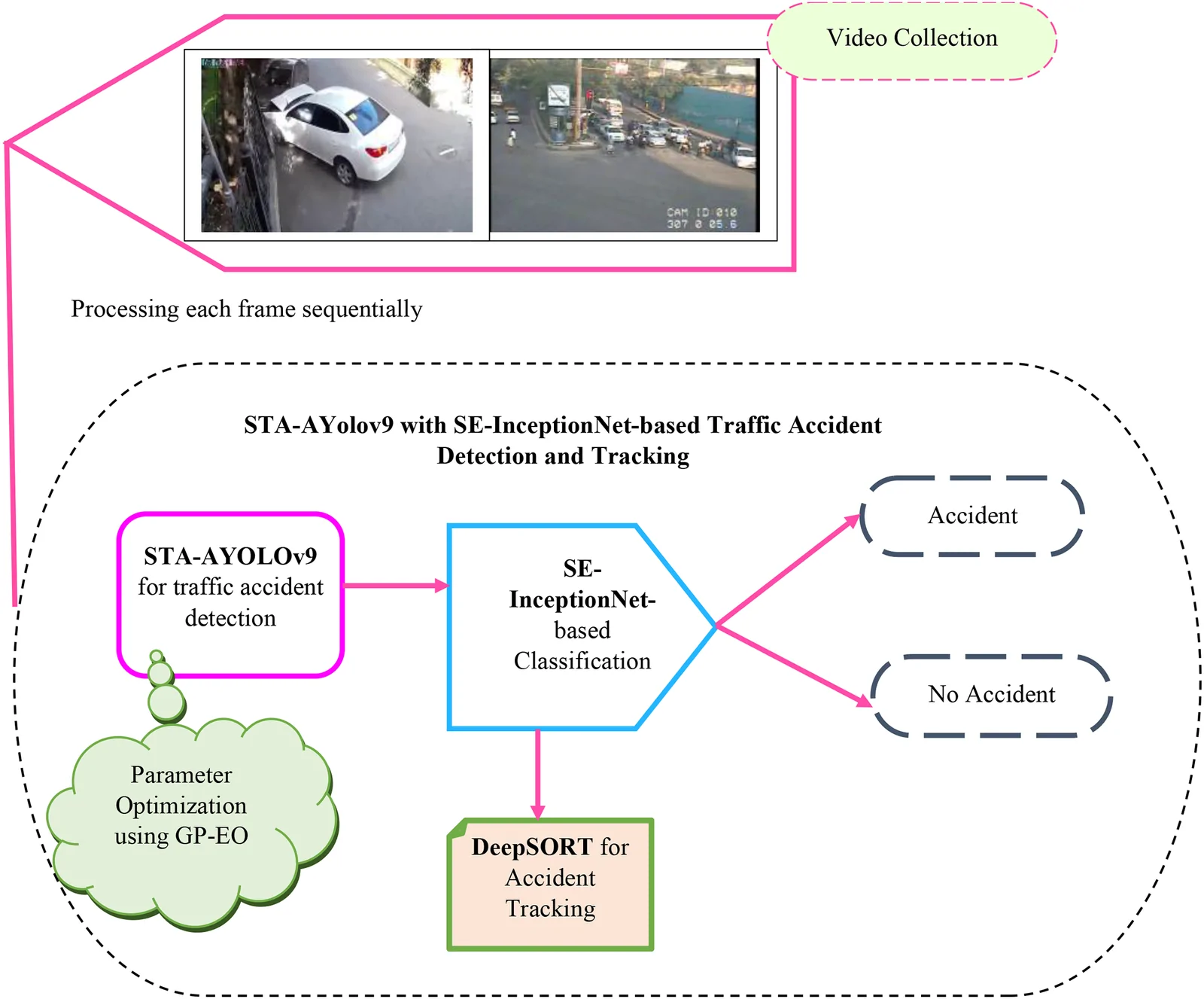
Structural illustration of the proposed model.

### Traffic surveillance video-based road accident detection and analysis

- **Task Definition:** This research aims to suggest an automated model for the detection and tracking of road accidents to encourage driver safety practices and improve traffic management. The suggested strategy is one of the efficient and accurate models for the timely recognition of road accidents in traffic surveillance. This model can significantly reduce the impact of accidents while improving rapid accident and emergency responses to improve the safety of the public. The adverse effects of accidents on public safety are ensured using the designed model, and it also improves emergency responses to increase the safety of the people. This research work mainly leverages deep learning architectures, such as spatio-temporal attention blocks, Yolov9, SE-InceptionNet and deep SORT for improving the response time to road accidents. Further, the road safety and lives of humans are saved by tracking road accidents, which ensures prompt assistance. Annually, numerous fatalities and injuries are caused by road accidents. The proposed model improves survival rates and minimizes the response time via the timely detection and tracking of road accidents using the suggested model. The number of accidents that happen each minute is increasing with the growing population. The accidents that occur on the road are unpredictable in nature. The nearby ambulances do not get the data about the accident situation. The lack of providing timely first aid services and the unavailability of medical services can lead to the loss of life of people in times. Using the recommended road accident prediction and tracking system, the time taken by the ambulance to reach the hospital can be greatly reduced, which helps to prevent loss of human life due to accidents. The road accident detection and tracking process is achieved by collecting the traffic surveillance video from open-source datasets. The key innovations of the developed traffic accident detection and tracking approach are given in the points below.
- **Traffic Accident Detection:** The YOLOv9 model is used to detect traffic accidents as quickly and accurately as possible. The YOLOv9-based traffic detection process involves the identification of specific accident events such as sudden stops, vehicle collisions, and vehicle behavior related to the accidents on urban streets, roads, and highways. YOLOv9 is highly effective in detecting traffic accidents when combined with an adaptive spatio-temporal attention module. It effectively recognizes the abnormal patterns, and hence the detection accuracy is greatly improved. The spatio-temporal attention module concentrates on spatial features and temporal features, which are how the objects move over time, which is important for detecting the sudden dynamics of accidents.
- **SE-InceptionNet-based road accident classification:** The traffic accident classification is effectively performed with the help of the developed SE-InceptionNet architecture that improves the feature selection performance and enhances the ability of the model by focusing on important spatial and temporal features. This model uses multiple filter sizes in parallel at each layer, thus allowing the model to capture a wide variety of features at various scales.
- **Deep SORT-based tracking:** After classifying the traffic accidents, the vehicles and persons involved in the incident are tracked using the developed Deep SORT model. The movement of objects is effectively tracked using this approach in complex scenarios such as interactions between multiple objects and occlusions. The deep SORT approach is effective at maintaining consistent tracking across frames, which ensures that the vehicles involved in an accident are continuously tracked.
- **Real-time traffic accident detection and tracking**: The integration of YOLOv9, spatio-temporal attention, SE-InceptionNet, and Deep SORT effectively performs real-time traffic accident detection and tracking. The exact location of the accident and the type of accident are effectively detected through the proposed model. The proposed model provides emergency response capabilities and also improves road safety. The suggested model adopts the IoT, so it is suited for effectively alerting the authorities when detecting accidents, which potentially saves human lives of humans. The severity label definition is shown in Table 2.

**Table 2:** Severity label definition.

Severity Class	Label Source	Visual Indicators Used	Interpretation
Fatal (High Severity)	Dataset annotation (heuristic)	High-impact collision, vehicle deformation, rollover, no motion after impact	Indicates potentially life-threatening scenario, not confirmed fatality
Injury (Moderate Severity)	Dataset annotation (heuristic)	Pedestrian/vehicle interaction, sudden stop, partial damage, human movement	Indicates likely injury scenario, not clinically verified
Property Damage (Low Severity)	Dataset annotation	Minor collision, low-speed impact, limited deformation	Indicates non-injury damage scenario
No Accident	Dataset ground truth	Normal traffic flow	No abnormal event
Clarification Table: Location & IoT Implementation			
Component	Current Work	Clarification/ Revision	Justification
Location Extraction	Implicitly assumed	Not derived from video alone	Video lacks GPS metadata
Geolocation Source	Not clearly defined	Requires external GPS/IoT sensors or camera metadata	Ensures realistic deployment
IoT Implementation	Conceptual	Defined as an architecture-level framework only	No physical deployment performed
Communication Protocol	Not specified	Added: MQTT/ HTTP-based communication	Standard IoT protocols
Deployment Validation	Not included	Clarified as future work	Avoids overclaiming
Exact Location Claim	Overstated	Revised to “approximate or externally provided location”	Scientifically accurate
Proposed IoT-Geolocation Pipeline (Added Explanation)			
Accident Detection (Vision Module)			• AYSIDSORT detects accident events from CCTV frames. • Camera-to-Location Mapping. • Each surveillance camera is associated with predefined GPS coordinates. • Location is retrieved from a camera registry database. • IoT Communication Layer.
Event data transmitted via			• MQTT protocol (lightweight, real-time) • HTTP REST API • Cloud/Server Layer
Stores:			• Accident timestamp • Camera ID • Associated GPS coordinates • Alert System
Sends notifications to			• Emergency services • Traffic management systems

### 3.3. Collection of traffic aerial images

This research uses the publicly available dataset for performing the road accident detection and tracking process.

***Dataset:*** The Accident Detection from CCTV Footage dataset is publicly available on the link https://www.kaggle.com/datasets/ckay16/accident-detection-from-cctv-footage with access date: 2026-6-30. The non-accident footage and accident footage from the CCTV cameras are included in this dataset. The accident in the real-time CCTV footage is determined using this dataset. The three folders of the dataset are train, test and val. The difference between the non-accidents and the accidents is determined by the consecutive frames included in this dataset. The video frames gathered from this dataset are given in Fig 3, and also the description of the datasets and experimental data preparation pipeline is shown in Table 3.

**Table 3:** Description of the datasets and experimental data preparation pipeline.

Aspect	Details
**Data Sources**	Publicly available datasets (e.g., traffic accident datasets) and curated open-source videos (e.g., YouTube, dashcam repositories)
**Dataset Type**	Real-world traffic surveillance and dashcam videos
**Train/Test Split**	Stratified split (e.g., 75%/ 25%) ensuring class balance
**Class Labels**	No Accident, Property Damage, Injury, High Severity (proxy-based labels)
**Annotation Method**	Manual annotation + validation based on visual cues (collision intensity, object interaction, motion patterns)
**Annotation Tool**	Standard tools (e.g., CVAT/LabelImg or equivalent)
**Language/Data Cleaning (if applicable)**	Removal of corrupted frames, irrelevant segments
**Access Information**	Links and references to datasets provided (or available upon request if licensing is restricted)
**Video-to-Frame Conversion**	
**Extract frames at fixed intervals using frame filtering**	It removes duplicate frames, blurry/low-quality frames, and non-relevant scenes
**Image standardization**	resize to fixed input size (e.g., 224 × 224)
**Data augmentation**	normalize pixel values, rotation, horizontal flip, brightness/contrast adjustment, label assignment
**Preprocessing steps**	Step-by-step pipeline provided

**Fig 3:**
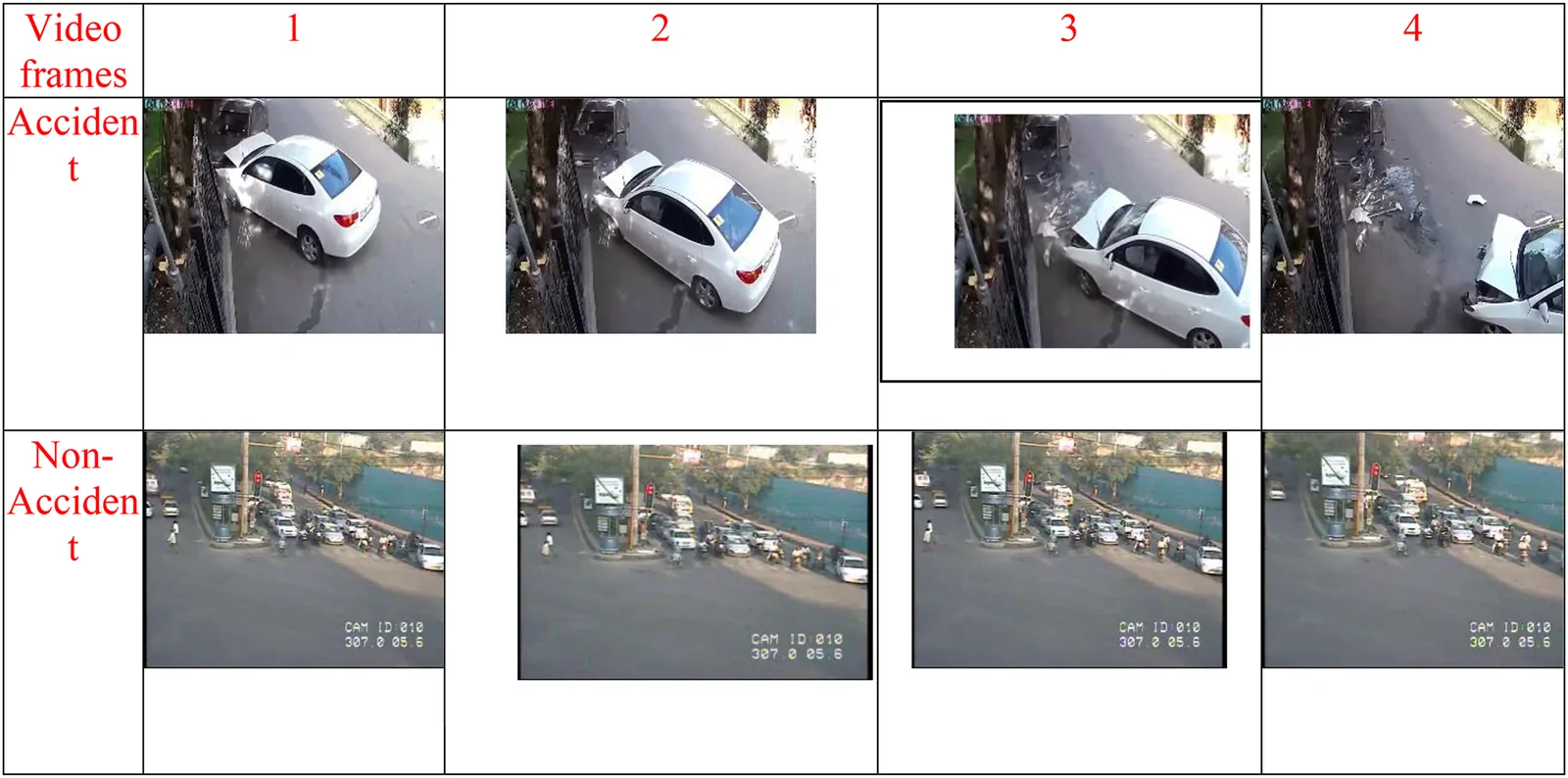
Video frames collected from the dataset.

## 4. Description of deep learning methods used for road accident detection and tracking

### 4.1. Spatio-temporal attention mechanism

The two attention modules, such as spatial and temporal attention, are the major components of the STA [37]. Here, the different weights are used to capture the data from the different tensors. The temporal attention layer is employed for representing all temporal modules, which is given in Eq. (1).


$$\begin{array}{c} sfu=\sum_{v=1}^{P_{`}}c_{1,v}\left(v,:,:,.\right) \end{array}$$ (1)

$$\begin{array}{c} c_{1,v}=\frac{Exp\left(\left(1-\chi_{\text{1}}\right)\cdot i_{v}\left(Y_{v}\left(v,;,;,.\right)\right)\right)}{\sum_{v=1}^{P_{\text{1}}}Exp\left(\left(1-\chi_{\text{1}}\right)\cdot i_{v}\left(Y_{v}\left(v,;,;,.\right)\right)\right)} \end{array}$$ (2)

The term $c_{1,v}$ expresses the weight of each temporal module, and the fully connected layer is specified as $i_{v}\left(\cdot \right)$, the temporal penalty factor can be used for managing the temporal attention’s function, and it is expressed as $Y_{v}:\chi_{\text{1}}\in \left[0,1\right]$, the term sfu expresses the output from the temporal attention layer. With the results of the temporal attention network, the spatial attention is applied on top of the temporal network, and it is specified in Eq. (3).


$$\begin{array}{c} \gamma^{c}=\sum_{u=1}^{P_{\text{2}}}c_{2,u}sfu\left(:,u,:\right) \end{array}$$ (3)

$$\begin{array}{c} c_{2,v}=\frac{Exp\left(\left(1-\chi_{\text{2}}\right)\cdot i_{v}\left(Y_{v}\left(v,;,;,.\right)\right)\right)}{\sum_{v=1}^{P_{\text{2}}}Exp\left(\left(1-\chi_{\text{2}}\right)\cdot i_{v}\left(Y_{v}\left(v,;,;,.\right)\right)\right)} \end{array}$$ (4)

The spatial network’s iweight is specified as$Y_{v}$, the output from the attention layer is represented as$\gamma^{c}$, the penalty factor is used for controlling the importance of the spatial attention, and it is expressed as$\chi_{\text{2}}\in \left[0,1\right]$.

### 4.2. Yolov9 Model

In terms of accident detection, the YoloV9 [38] provided improved accuracy and reduced computing complexity. The addition of these modules minimizes the amount of data lost during person tracking. The accident detection accuracy and the robustness of the person detection and tracking system are increased via the augmentation within this model.

*Backbone*: The YoloV9 model uses GELAN to perform multiscale feature extraction, just like the YoloV8 model. The residual blocks, dense block and CSP blocks are the essential computing blocks in the GELAN module. The hierarchical properties throughout all layers of the models are kept by these blocks. The PGI integrated with the YOLOV9’s neck architecture boosts the feature aggregation process of the model. The feature fusion from diverse layers is improved by integrating the PGI with the multilevel auxiliary data.

*Head*: The reversible detection facilitated by the PGI is utilized in the YoloV9 model to achieve better detection without anchors. It gives fewer computational resources during the accident prediction, and preserves all data during both forward and backward predictions. The structural demonstration of the Yolov9 is provided in Fig 4.

**Fig 4:**
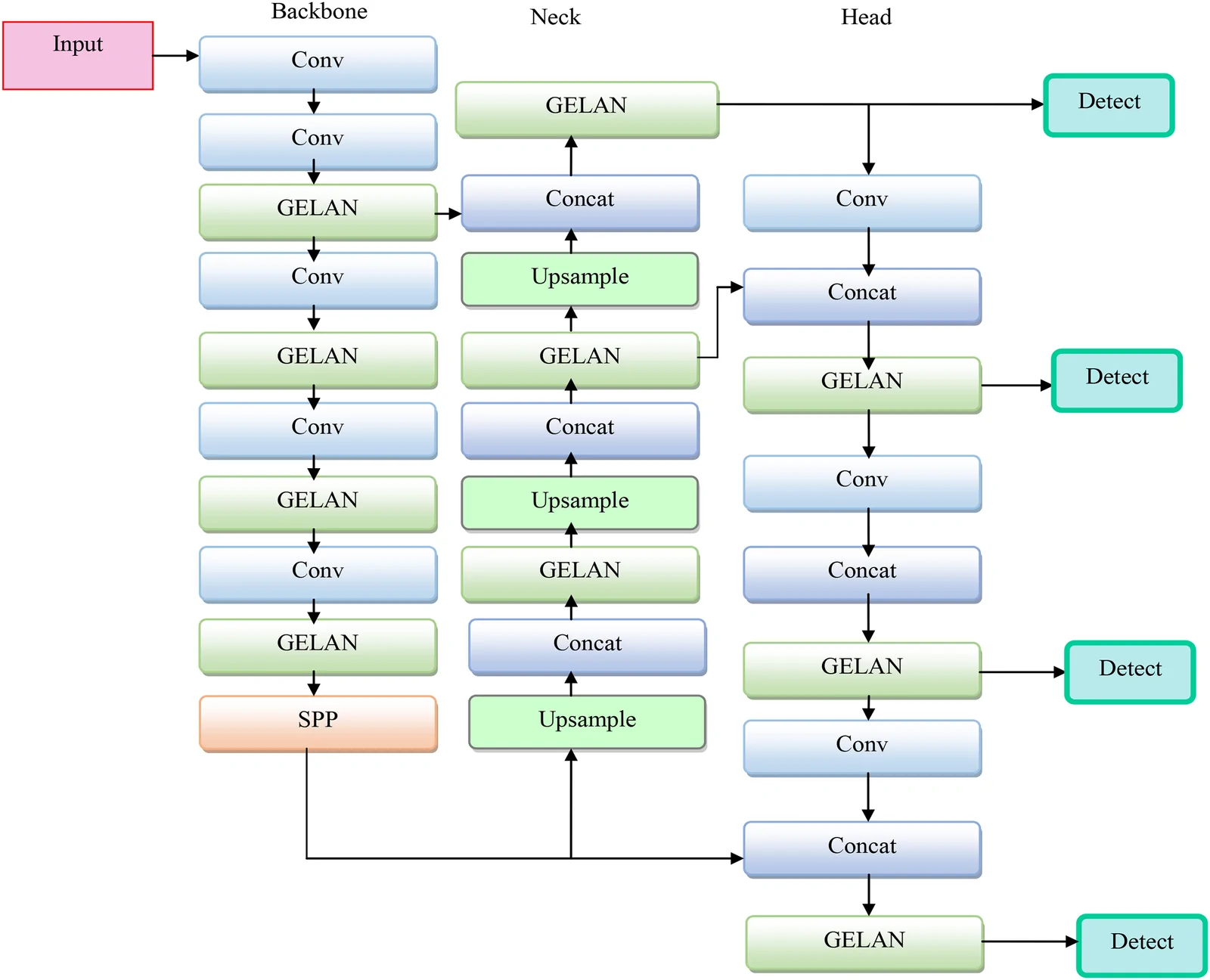
Pictorial Illustration of the Yolov9 Model.

### 4.3. SE-InceptionNet Model

The SE attention improves the performance of the accident detection [39] by suppressing the non-critical zone and focusing on the significant units of the images, because of the spatial correlation extracted by the filters and the inter-channel dependencies of the convolutional output. The SE attention block increases the volume of the transportation activity. The reduction ratio at the excitation step is used to build the first fully connected layer with the ReLU activation.

The InceptionNet [40] is a sophisticated model, and here the depth of the model is expressed by the term inception. The issues raised by the deep learning models are addressed by the Inception technique. Because it mimics the vanishing gradient problems and computational requirements during the detection task, the InceptionNet model achieved remarkable accuracy in skin cancer detection. The filter sizes 5×5,3×3,1×1are present in the single layer of the InceptionNet. The pooling activation layer, batch normalization layer, and dense layer of InceptionNet are responsible for learning the complex patterns in the images to allow efficient detection. Some layers in this network efficiently learn the complex correlations found in the images. The structural diagram of the SE-InceptionNet model is provided in Fig 5.

**Fig 5:**
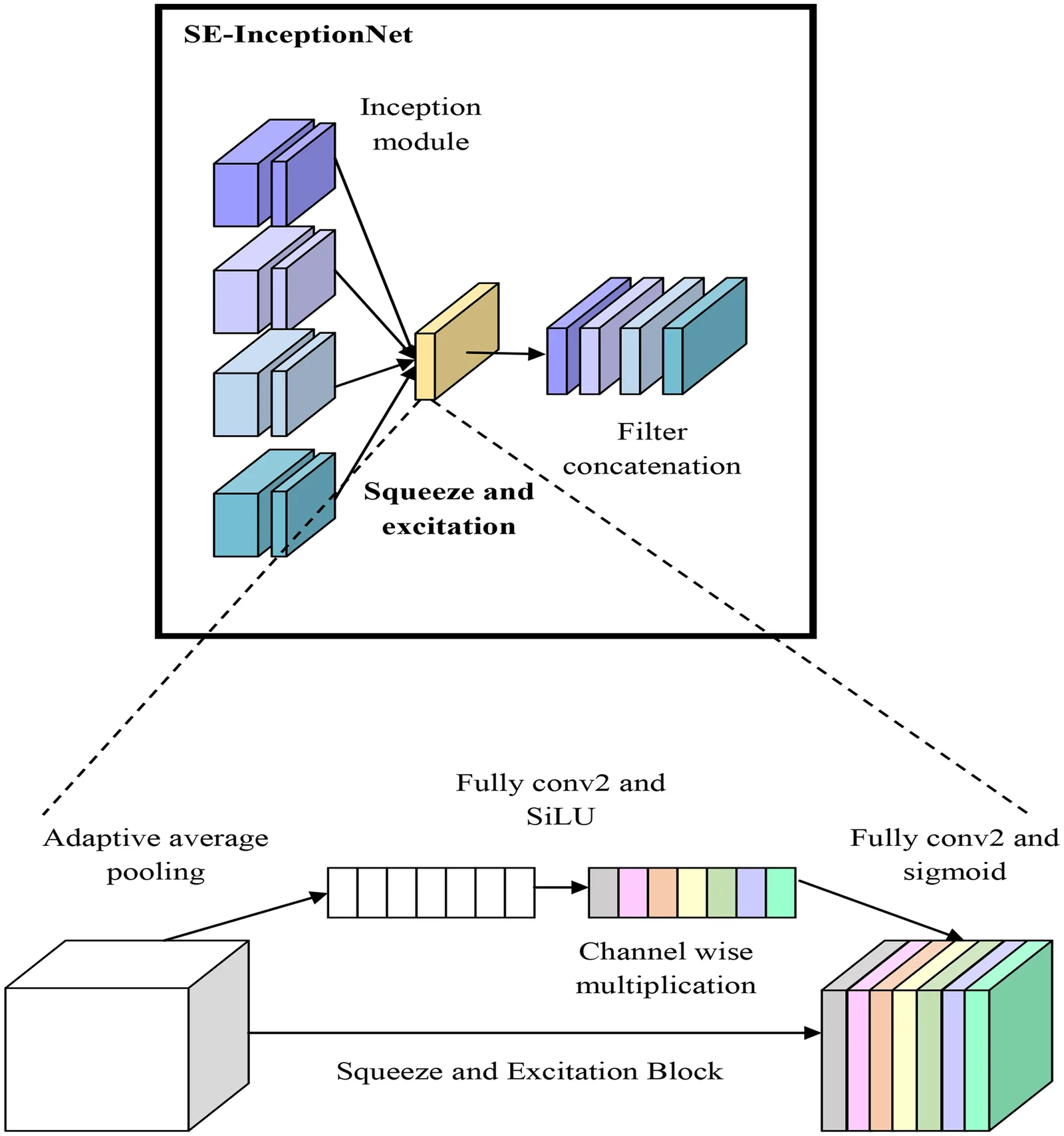
Pictorial Illustration of the SE-InceptionNet Model.

### 4.4. DeepSORT Model

***Description of DeepSORT model:*** Real-time object tracking is done with the support of the DeepSORT [41] model which is utilized to preserve the identity and trajectory of vehicles over consecutive frames in road surveillance videos. In road accident detection systems, the Deep SORT is incorporated after a vehicle detection network to track each vehicle involved in sudden stops, abnormal motion patterns, and normal traffic movement. It expands the baseline SORT algorithm by integrating deep appearance features, which enhances the tracking robustness during crowded traffic conditions.

**Working Principles of DeepSORT Model:** The working principle of Deep SORT in a road accident monitoring framework includes four major phases that are depicted as follows.

- **Phase 1: Vehicle Detection and Initialization**

At the initial stage, each video frame attained from the CCTV surveillance system is handled by an object detector to recognize road users such as motorcycles, trucks, cars, and buses. Each detected vehicle is assigned a unique track identity (ID), and its position is upgraded in subsequent frames.

- **Phase 2**: **Motion Prediction using Kalman Filter**

After that, Deep SORT uses a Kalman Filter approach to validate the future position of each tracked vehicle according to its prior motion characteristics. This Kalman Filter efficiently predicts the next location of the vehicle when the vehicle is in a hidden state owing to occlusion and accident impact.

- **Phase 3: Deep Appearance Feature Extraction**

The baseline SORT approach mainly depends on motion information, but the Deep SORT captures visual appearance features with the support of a deep Convolutional Neural Network (CNN). It transforms each vehicle image region into a high-dimensional feature vector. It computes similarity between detected vehicles and baseline tracks.

- **Phase 4: Data Association using Hungarian Algorithm**

The suggested Deep SORT model combines motion information and appearance similarity to associate present detections with baseline vehicle tracks. Hungarian optimization algorithm chooses the best matching between predicted vehicle positions and new detections.

The feature appearance is computed using a pre-trained CNN model. The architecture of the DeepSORT model is given in Fig 6.

**Fig 6:**
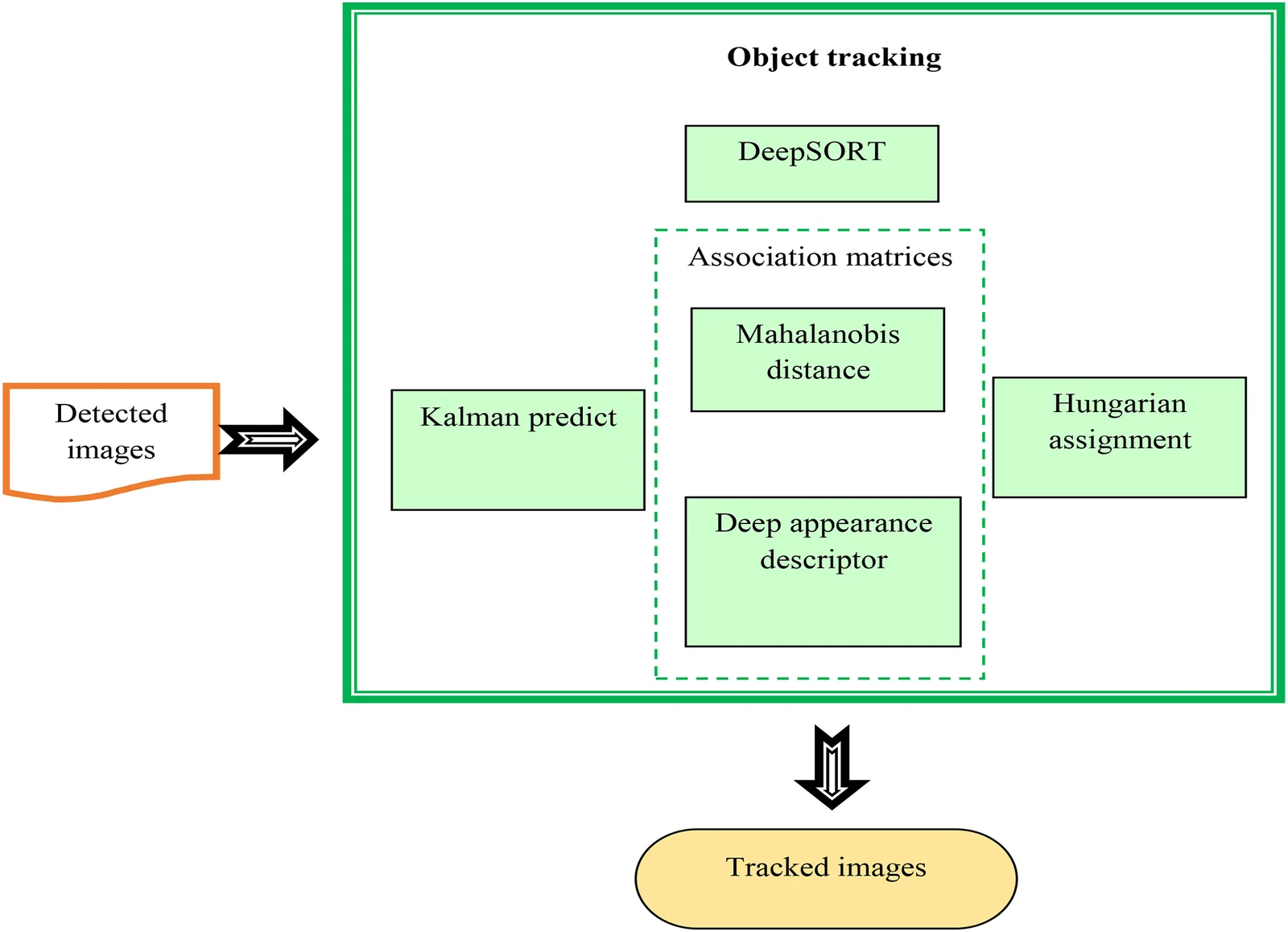
DeepSORT for Accident Tracking.

## 5. IoT-oriented road accident detection and tracking framework using an advanced deep learning network

### 5.1. Designed GP-EO

The GP-EO is used for optimizing the features of the detection task. The optimal feature and weight selection are used for improving the overall efficiency of the road accident recognition and tracking approach by reducing the variance and generalization error. To achieve optimal performance in road accident detection and tracking, optimizing the parameters is an important process. The nonlinear functions are also more effectively solved using the GP-EO. Road accident prediction is more effectively performed using the GP-EO since it optimally selects the features along with the weights instead of random selection. None of the existing approaches adopts the improved version of the algorithm for assigning the weights, so this research proposed GP-EO, which is the improved version of the EO for optimal parameter selection. The GP-EO is conceptualized from the EO for providing optimal solutions. The execution process of the GP-EO is very simple; it also requires less tuning of parameters and early convergence. The potential solution to the problems to be solved are represented by the individual candidates in the proposed GP-EO. In addition, the GP-EO guides the optimization towards the direction of determining the optimal solutions. The integration of the GP-EO in the optimal weighted feature fusion endeavours to improve the accuracy of road accident recognition and tracking. The redundancy and computational cost of the road accident detection are reduced by the GP-EO optimization. The GP-EO effectively explored the search space for improving the generalization efficiency of the parallel cascaded model. The EO [42] mimics the control volume mass balance models to determine the best possible solutions. Various optimization problems are efficiently using the EO, and it is used for solving the economic dispatch problems. But the EO exhibit a tendency to local optima, and the imbalance between the exploitation and the exploration issues significantly affects the performance of the EO. Most importantly, the susceptibility to the loss of population diversity is the greatest issue in the EO. To alleviate those issues, the random number of the EO is upgraded by considering the current, mean and best fitness values. The formula for calculating the new random variable is provided in Eq. (5).


$$\begin{array}{c} y=\frac{wr+et}{qw+ty} \end{array}$$ (5)

Here, the current and best fitness are represented as wr,et and the mean fitness and worst fitness are specified as qw,ty. The enhanced random number is represented as y. The imbalance between exploration and exploitation issues is effectively solved using the improved random number. In addition, the loss in population diversity is also more effectively addressed using the GP-EO. The pseudocode of the GP-EO is provided in Algorithm 1.

Algorithm 1. GP-EO

**Input**: Features and weights

**Output**: Optimized features and optimized weights

Fix the population and iteration count

Initialize the population function and evaluation

While the termination is not fulfilled

Find the equilibrium pool and the candidate solutions.

Determine the value of the generation rate

**Determine the new random number using Eq. (5)**

Find the particle’s memory-saving

Update the concentrations

End while

Get best solution

Stop

### 5.2. Implemented AYSIDSORT for Road Accident Detection and Tracking

Road accident detection and tracking is one of the imperative operations for improving road safety. In the realm of autonomous driving, reliable environmental perception is achieved via the AYSIDSORT-based road accident detection and tracking. In this network, the performance of the road accident detection and tracking is enriched via the integration of YoloV9, STA, SE-based InceptionNet and Deep SORT. In the given input frame, the objects in the frame are accurately determined and classified through the adaptive YOLOV9 model. Then, Deep SORT processes those entities for the tracking process. The scenarios involving light and dense traffic variation are more effectively handled by this model. In addition, in the complex real-world situation, more effective and promising navigation is achieved via the suggested AYSIDSORT model. In this model, the computational efficiency is optimized by the YOLOV9 model and the loss of data is also prevented by the adaptive concept within the YOLOV9 model. Initially, the input frames are passed to the Spatio-Temporal Attention-based Adaptive Yolov9, where it detects the object in each frame via the bounding boxes. The accidents are effectively classified using this YoloV9 model with a Spatio-Temporal attention module. The SE-InceptionNet model classifies traffic accidents to provide emergency responses. In addition, the channel-wise feature responses are recalibrated by the SE block, which helps to focus on the more informative features from the image frames. Moreover, features at various scales are captured by the inception modules so that the objects of various scales are more effectively captured by the proposed model. For each detected object, the discriminative representations are extracted by the SE and the inception block within the system. The consistent object identities across multiple detections are maintained by the deep SORT algorithm. In this model, deep SORT is used for providing a complete tracking solution by taking the decision from the Yolov9 model. Real-time object detection and tracking are more effectively done with the integration of YOLOV9 and Deep SORT. Here, the pipeline named AYSIDSORT is used for achieving a robust solution for road accident detection and tracking. Here, higher detection capabilities are provided by the YOLOV9 model, and consistent tracking is provided by the Deep SORT structure. The combination of Deep SORT and YOLOV9 model provides higher detection speed and better accuracy in accident detection and tracking. In this model, the parameters of the Spatio-Temporal Attention-based Adaptive Yolov9 with SE-Inception Net are tuned using the GP-EO to get better results in object detection and tracking. The steps of the AYSIDSORT-based road accident detection and tracking are given as follows:

The procedure below is done for each frame $G_{u}\text{\textbackslash{}hspace\{0.17em}\;\}in\text{\textbackslash{}hspace\{0.17em}\;\}\left\{G_{\text{1}},G_{\text{2}},..G_{o}\right\}$.

***Step 1***: Accident detection:- The object detector is initially run, and it is expressed as $E_{u}\text{\textbackslash{}hspace\{0.17em}\}\left|G_{u}\right|$. The bounding box bj, class label $m_{j}$, and confidence score dj are taken for each detection ej in $E_{u}$.

***Step 2***: Appearance feature extraction:- The appearance embedding is computed for each detection ej, and it is expressed in Eq. (6).


$$\begin{array}{c} ej\leftarrow FeatureNet\left(Crop\left(G_{u},c_{j}\right)\right) \end{array}$$ (6)

***Step 3:*** Motion prediction:- The current state is predicted for each existing track $U_{j}\in U$, and it is expressed in Eq. (7).


$$\begin{array}{c} Uk.state_{u}\leftarrow Kpredict\left(U_{j}.state_{u}\right) \end{array}$$ (7)

**Step 4**: Data association;- For the matching track and detection, the cost matrix Dis computed and it is determined using Eq. (8).


$$\begin{array}{c} \begin{array}{c} D\left[j\right]\left[k\right]=϶\times Mahalanobisdist\left(U_{j}.state,c_{j}\right)\\ +\left(1-϶\right)\times cosinedist\left(uj\cdot getthematchedfeature\right) \end{array} \end{array}$$ (8)

***Step 5:*** Track Update:- The appearance embedding $U_{j}\cdot feature\leftarrow g_{j}$ is updated for each matched pair $\left(U_{j},e_{j}\right)$.

***Step 6***: Track management:- The age of the track is increased for the unmatched track, and if the predefined thresholds are exceeded, then the unmatched track is removed. With the help of the feature representation, the new unique track ID is allocated.

***Step 7***: With the current object identities and states, the updated track set is returned. The structural illustration of the AYSIDSORT for road accident detection and tracking is illustrated in Fig 7.

**Fig 7:**
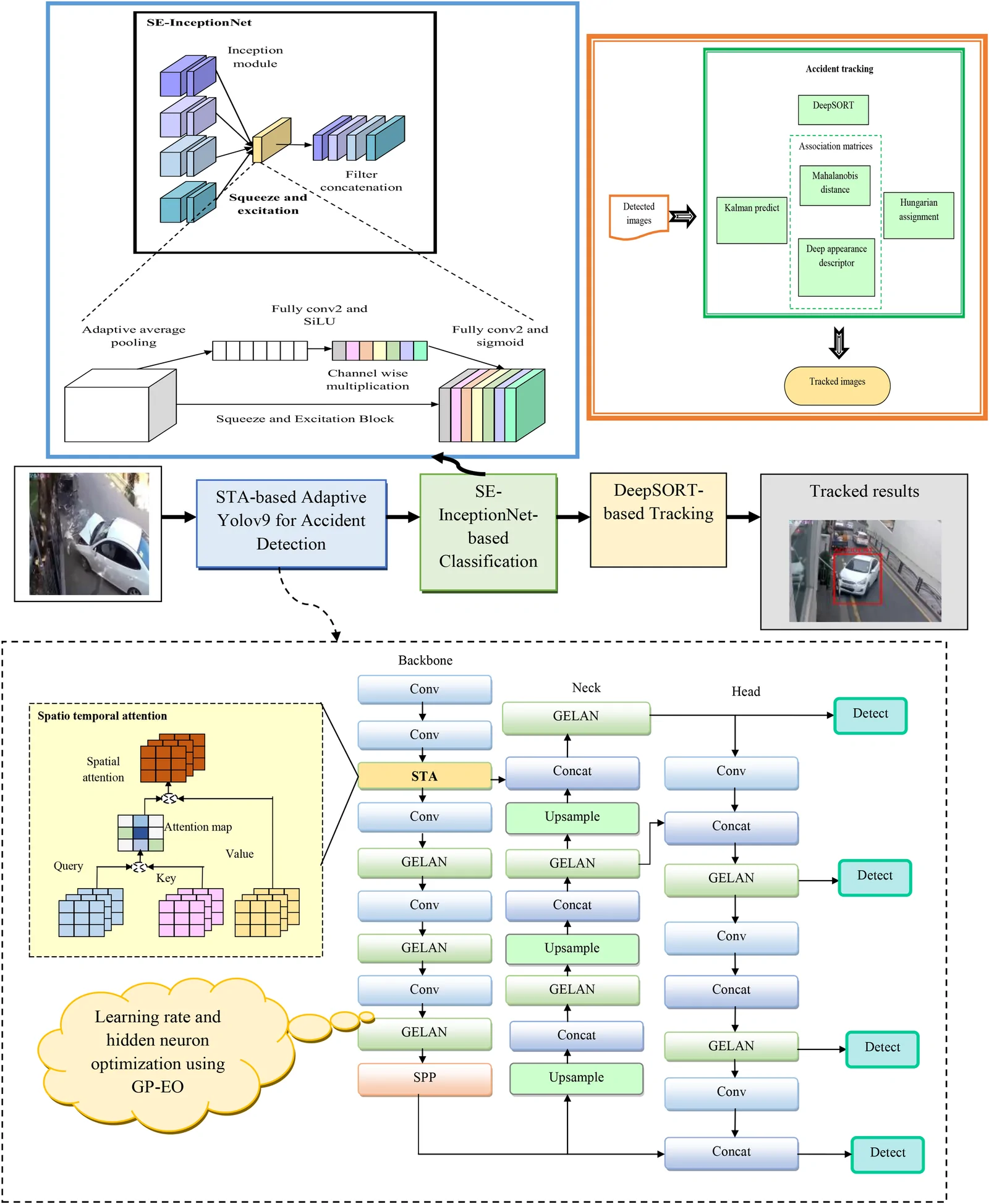
Structural view of the proposed approach for road accident detection and tracking.

## 6. Results and discussion

### 6.1. Experimental environment

The proposed road accident detection and tracking model is implemented using Python platform. The training parameters, such as number of population size, was taken as 10, the iteration count as 50, and the chromosome length as 44 for validating the performance of the recommended road accident detection and tracking model. The Orangutan Optimization Algorithm (OOA) [43], Crisscross Optimization Algorithm (COA) [44], Flower Fertilization Optimization (FFO) [45], and EO [42], Seq2Seq-LSTM-AE [24,46], YoloV3 [25,47], Fuzzy vision-based DNN [28,48], MLP (without optimization) were utilized for validating the performance of the presented road accident detection and tracking approach. The GPU hardware details of this suggested approach is shown in Table 4.

**Table 4:** GPU hardware details of the suggested approach.

GPU	NVIDIA RTX 3080 (10GB)
CPU	Intel i7 Processor
RAM	16 GB
Learning rate schedules	
Initial Learning Rate	0.001
Optimizer	Adam
Scheduler	Step decay
Total Epochs	50
Number of populations	10
Iteration count	50
Chromosome length	44
Data augmentation strategies	No augmentation
Confidence Threshold (Detection)	
Confidence Threshold	0.5
Non-Maximum Suppression (NMS)	0.45
IoU Threshold (Tracking Association)	
IoU Threshold	0.5
Matching Strategy:	Hungarian algorithm with appearance + motion cues (DeepSORT)

### 6.2. Performance indices

The terms Accuracy, Dice coefficient and IoU are utilized for ensuring the effectiveness of the proposed approach.


$$\begin{array}{c} accuracy=\frac{Tp+Tn}{Tp+Tn+Fp+Fn} \end{array}$$ (8)

$$\begin{array}{c} dice=\frac{2Tp}{2Tp+Fp+Fn} \end{array}$$ (9)

$$\begin{array}{c} IoU=\frac{Tp}{Tp+Fp+Fn} \end{array}$$ (10)

Here, the terms Tp,\hspace{0.17em}and\hspace{0.17em}Tn denote the true positives and negatives, and the terms Fp,\hspace{0.17em}and\hspace{0.17em}Fn express the false positives and negatives.

### 6.3. Tracked images

The tracked images from the traffic surveillance video are listed in Fig 8.

**Fig 8:**
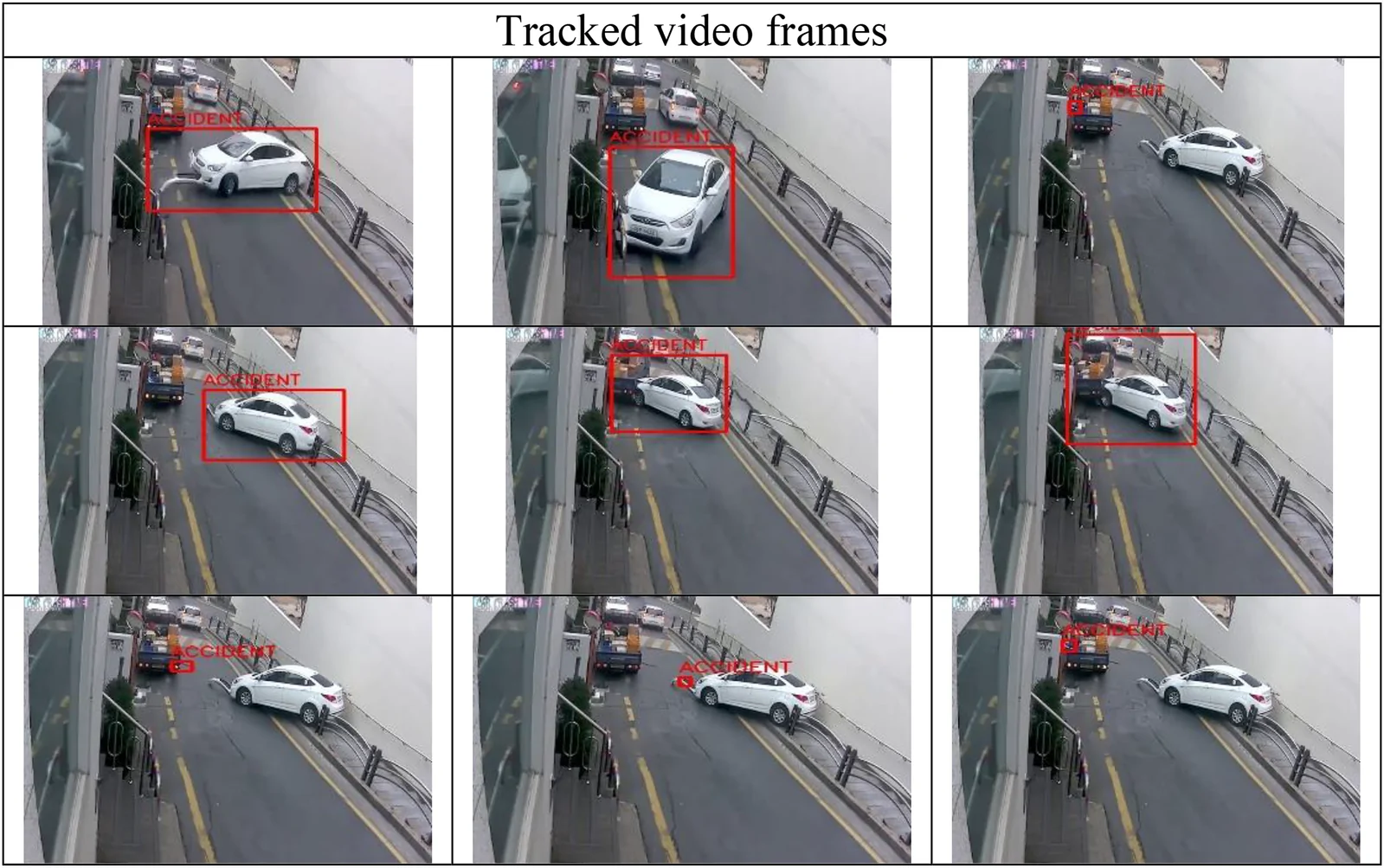
Tracked images from the proposed model.

### 6.4. Convergence validation of the suggested approach

The experimental results of the suggested GP-EO with conventional algorithms are given in Fig 9, which demonstrates the convergence ability of the suggested GP-EO and its effects in the road accident detection and tracking. This is the straightforward comparison for the proposed GP-EO in determining its performance in road accident detection and tracking. From the convergence analysis results, it can be seen that the suggested GP-EO provides an earlier convergence rate than the other algorithms, such as COA-AYSIDSORT, FFO-AYSIDSORT, OOA-AYSIDSORT, and EO-AYSIDSORT. Here, the suggested GP-EO provides better convergence and also converges with a very small number of iterations. The rapid convergence of the suggested GP-EO proved that the algorithm can effectively balance the phases of exploitation and exploration to get the best results. The most effective parameter searching ability of the developed GP-EO approach is validated with the iteration-based minimized cost function values.

**Fig 9:**
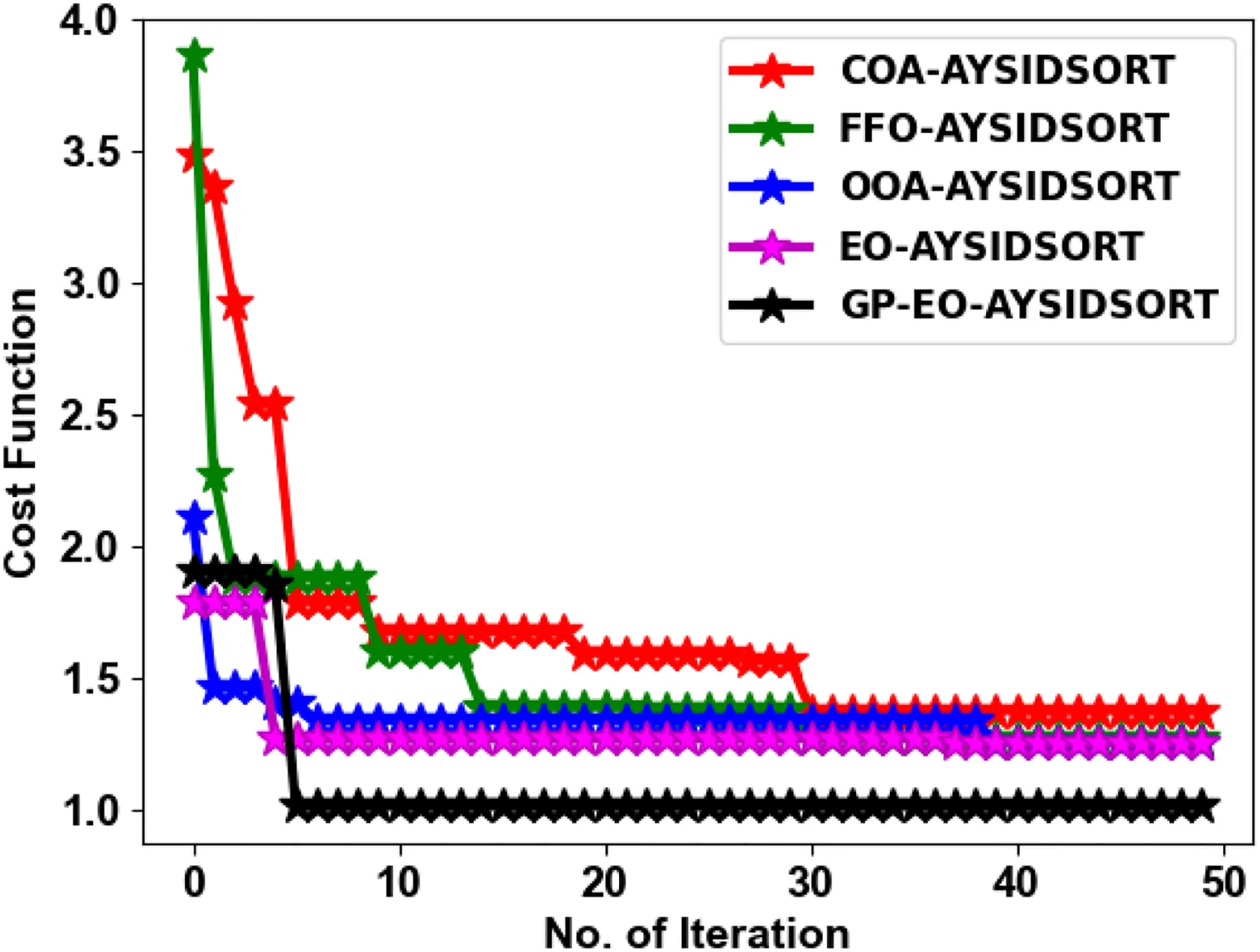
Convergence validation of the presented road accident detection and tracking approach.

### 6.5. Accident detection performance validation

The performance of the suggested AYSIDSORT in the accident detection is validated with various metrics across the previous works, and the optimization methods are showcased in Fig 10 and Fig 11. Batch size variation-based performance validation is helpful to effectively analyze the traffic accident detection performance. Examining the results from the graph below helps to comprehensively assess the performance of the designed AYSIDSORT in road accident detection. By carefully examining the results, the accuracy of the suggested AYSIDSORT reaches to 95% in the 100^th^ epoch, whereas there is a slight variation in the accuracy values when the batch size is varied from 4 to 128. The dice coefficient and IoU-based performance validation ensure the effective traffic detection performance across the traditional works. Thus, the accurate results from the proposed AYSIDSORT are used for managing the transportation and also lead to substantial improvements in the road transportation system. Here, the proposed GP-EO-AYSIDSORT models combine the two machine learning models in parallel to reduce the accident rate and the adverse effects on business and human health.

**Fig 10:**
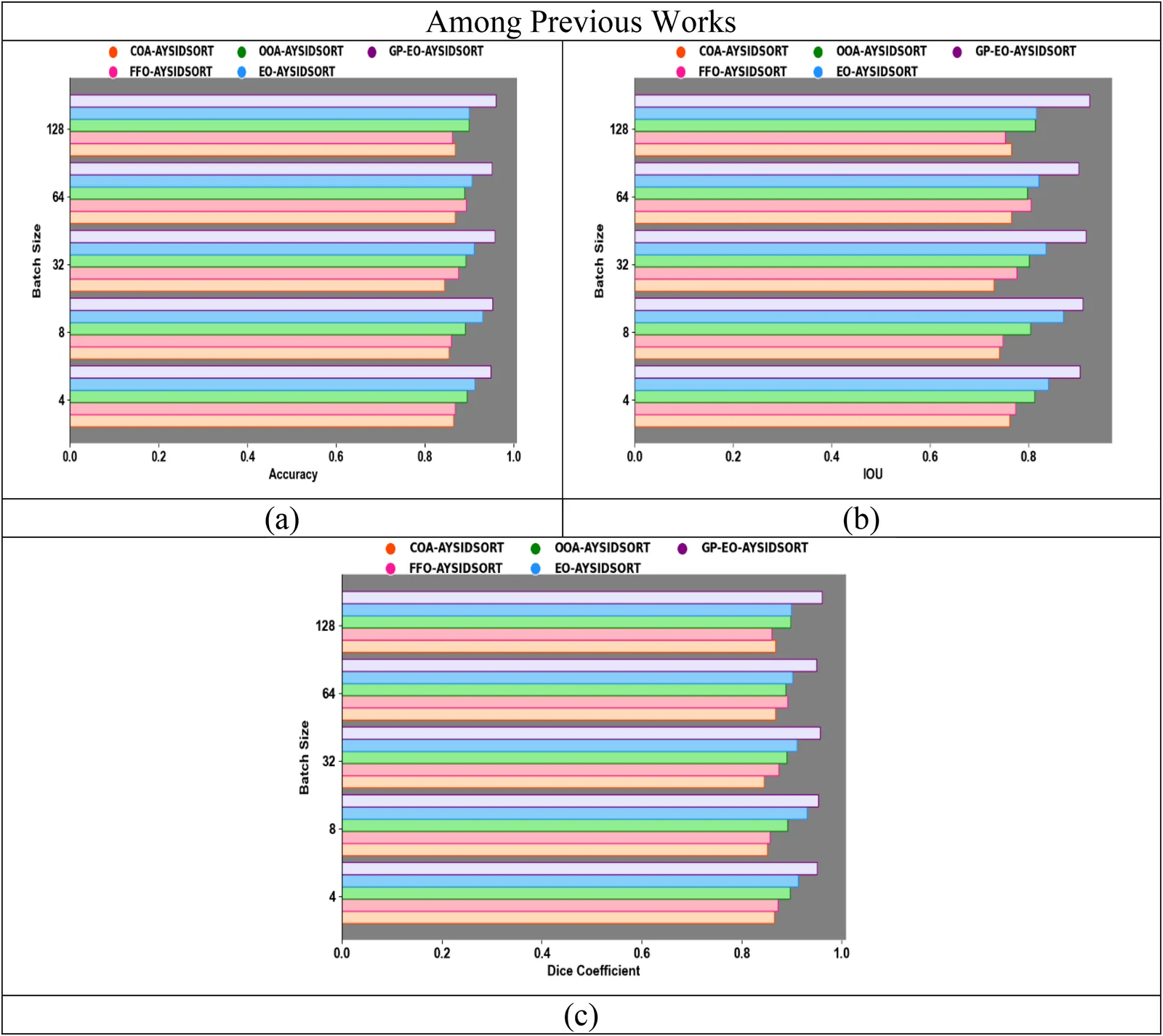
Accident detection efficiency of the offered framework regarding (a) Accuracy, (b) IoU, and (c) Dice Coefficient.

**Fig 11:**
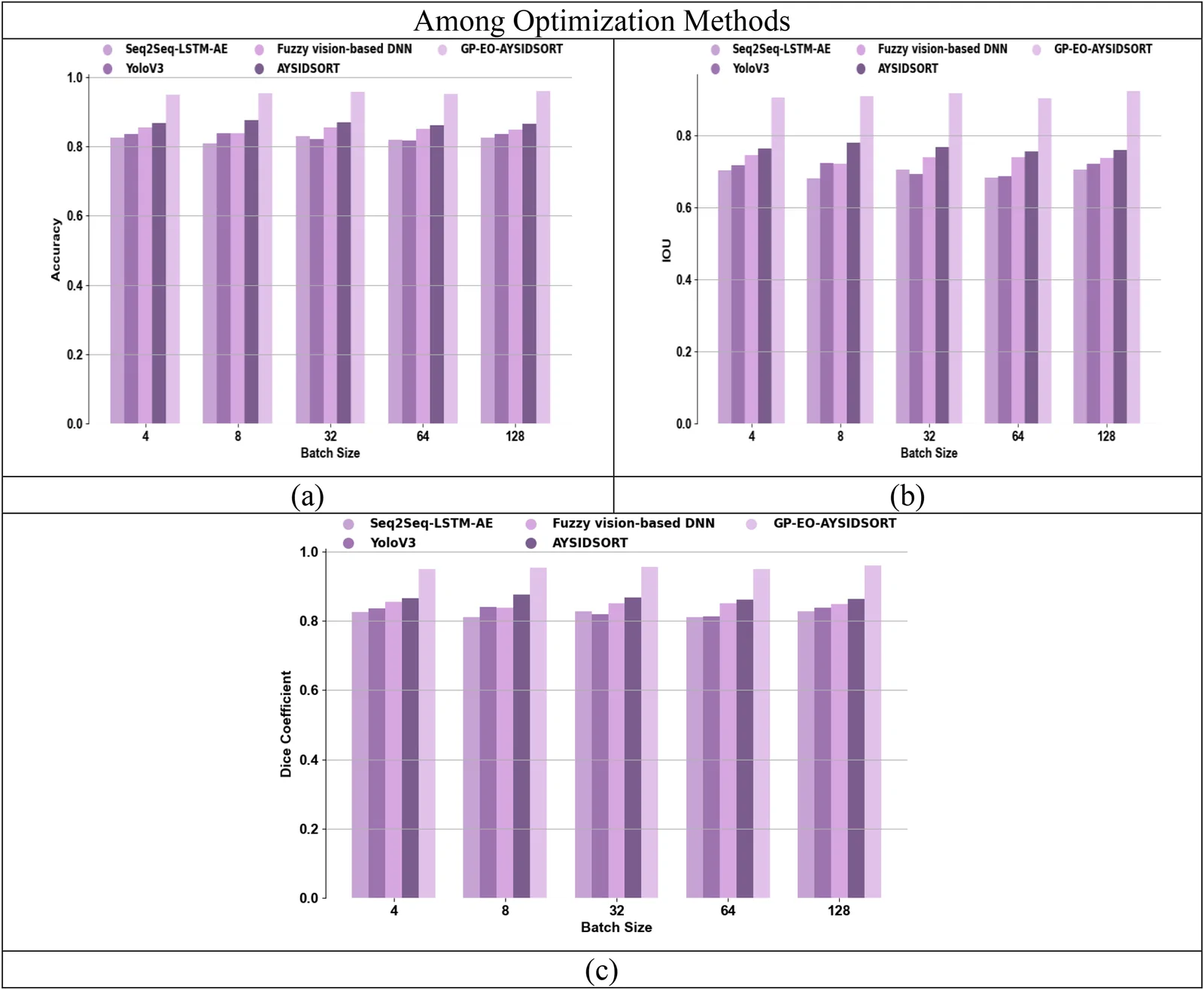
Accident detection performance of the suggested model regarding (a) Accuracy, (b) IoU, and (c) Dice Coefficient.

### 6.6. Accident classification performance

The results of the developed traffic accident classification performance of the developed GP-EO-AYSIDSORT model are provided in Fig 12. Some traditional accident classification approaches are considered for the validation process, and the results confirmed that the suggested approach consistently achieves the highest scores in all cases, which indicates their superior ability in classifying traffic accidents. Among all the optimizers used for the validation, the Adam optimizer achieves the most effective performance, rather than the optimizers such as SGD and Adagrad.

**Fig 12:**
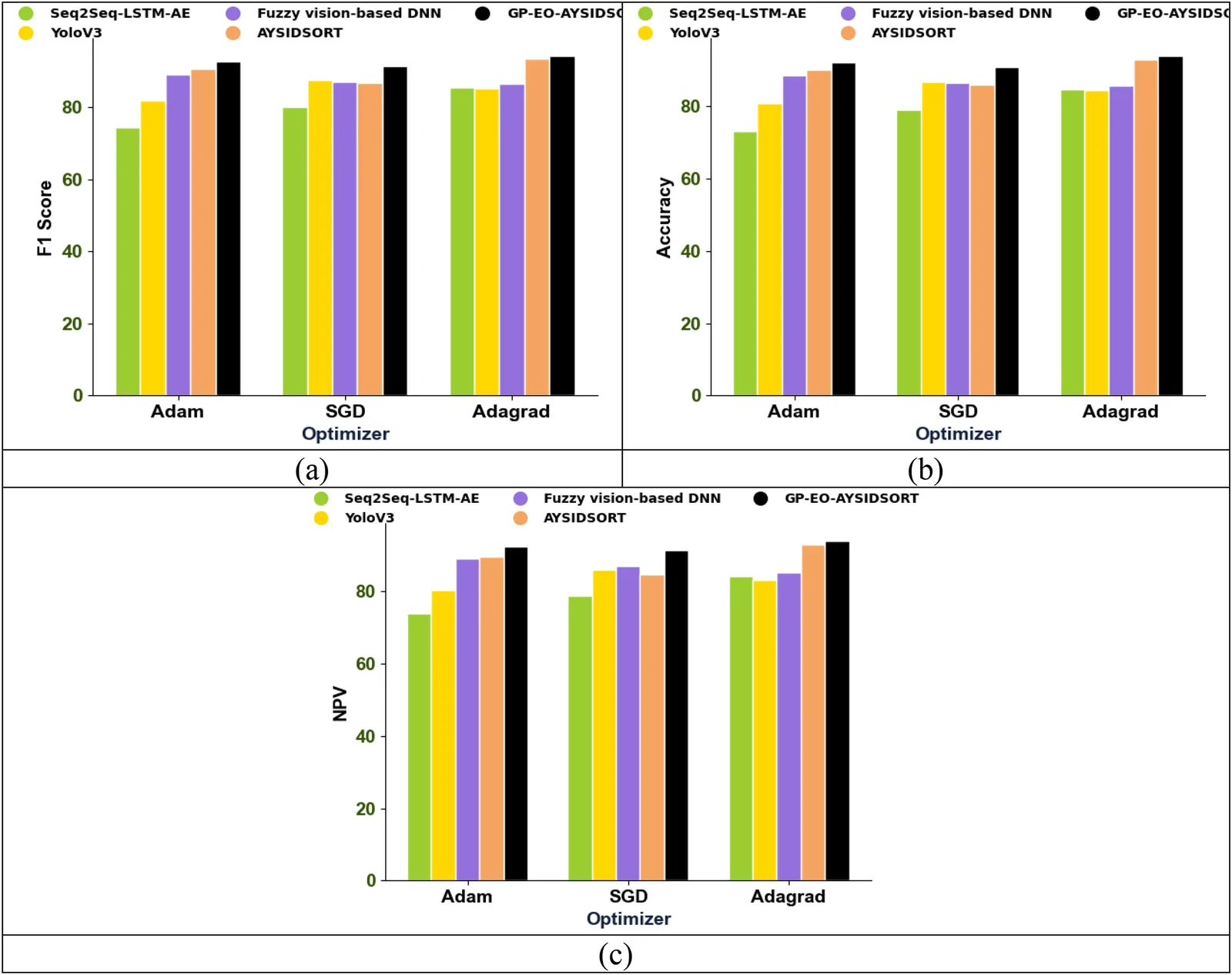
Optimizer-based analysis of the presented road accident detection model for (a) F1-score, (b) Accuracy and (c) NPV.

The YOLOv3 model performs well compared to AYSIDSORT and GP-EO-AYSIDSORT while using measures like F1 score and accuracy. The Seq2Seq-LSTM-AE and Fuzzy Vision-based DNN models achieve lower performance among all metrics when compared to the YOLOv3 model. However, the designed GP-EO-AYSIDSORT obtains more effective performance regarding measures like accuracy, F1-score, and NPV, which shows the effective real-time traffic accident classification performance of the designed approach.

### 6.7. Performance analysis over accident tracking

The analysis in Fig 13 indicates the tracking performance of the proposed AYSIDSORT over various metrics. The training time analysis of the presented approach ensures the lowest time required for detecting real-time accidents, which is 6.2 seconds, which is lower when compared to the baseline accident tracking methods. The mean reward, maximum reward and minimum reward analyses ensure effective real-world tracking performance while analyzing complex environments. The accident tracking performance of the designed AYSIDSORT model is more accurate than the baseline techniques, such as Seq2Seq-LSTM-AE, Yolov3, Fuzzy vision-based DNN and AYSIDSORT approaches. The suggested AYSIDSORT can provide a warning even in the case of high-risk traffic accidents.

**Fig 13:**
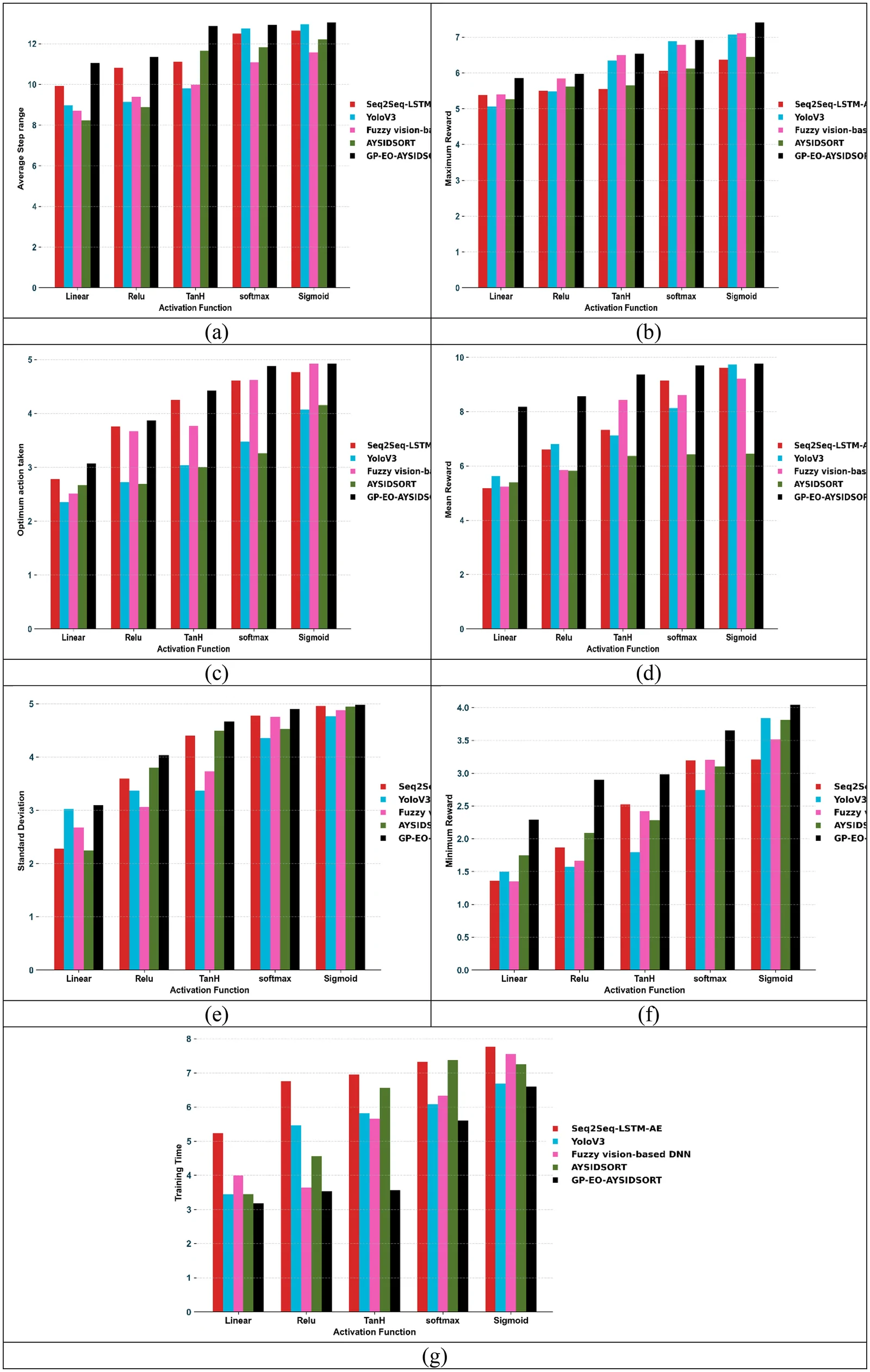
Proposed road accident tracking performance concerning measures like (a) Average step range, (b) Maximum reward, (c) Optimum action taken, (d) Mean reward, (e) Standard deviation, (f) Minimum reward and (g) Training time.

### 6.8. Numerical results of road accident detection and classification process

In Table 5 and Table 6, the accuracy of the suggested AYSIDSORT -based road accident detection and tracking model is validated by varying the learning percentage in the range of 35–75. Here, the proposed model is validated based on statistical metrics such as best, worst, mean, median, and standard deviation, which are defined as follows.

**Table 5:** State-of-the-art analysis of the suggested road accident detection and tracking approach.

TERMS	COA- AYSIDSORT [44]	FFO- AYSIDSORT [45]	OOA- AYSIDSORT [43]	EO- AYSIDSORT [42]	GP-EO- AYSIDSORT
**Accuracy**					
Best	0.871821	0.892309	0.907838	0.922473	0.9412
Worst	0.840843	0.867458	0.873139	0.887447	0.912401
Mean	0.858602	0.877775	0.891104	0.908437	0.926262
Median	0.856644	0.87778	0.895203	0.911896	0.925295
Standard deviation	0.009161	0.006951	0.010719	0.010888	0.008457
Dice coefficient					
Best	0.772768	0.805558	0.831231	0.856103	0.888931
Worst	0.725392	0.765938	0.774842	0.797667	0.838914
Mean	0.75235	0.782243	0.803764	0.832416	0.862768
Median	0.749238	0.782184	0.810287	0.838059	0.860976
Standard deviation	0.014048	0.011074	0.017375	0.018134	0.014654
Jaccard					
Best	0.871552	0.892807	0.906921	0.923416	0.941483
Worst	0.840622	0.863815	0.876175	0.891739	0.912491
Mean	0.858456	0.877905	0.891997	0.909189	0.926552
Median	0.858078	0.878304	0.894768	0.912277	0.926353
Standard deviation	0.009336	0.008241	0.00947	0.009945	0.008505
TERMS	**YOLOv8 [49]**	**YOLOv10 [50]**	**Fuzzy vision-based DNN [28]**	**AYSIDSORT**	**GP-EO-AYSIDSORT**
**Accuracy**					
Best	0.825984	0.844103	0.854473	0.883358	0.9412
Worst	0.797026	0.818634	0.828046	0.849142	0.912401
Mean	0.814979	0.831086	0.845144	0.864012	0.926262
Median	0.818847	0.832289	0.845025	0.862016	0.925295
Standard deviation	0.010537	0.008819	0.007224	0.01015	0.008457
Dice coefficient					
Best	0.703554	0.730258	0.745921	0.791084	0.888931
Worst	0.662546	0.692955	0.706552	0.737835	0.838914
Mean	0.687866	0.711087	0.731885	0.760723	0.862768
Median	0.693264	0.712754	0.731639	0.757494	0.860976
Standard deviation	0.014947	0.01292	0.010764	0.015804	0.014654
Jaccard					
Best	0.826279	0.843277	0.854813	0.882233	0.941483
Worst	0.800079	0.817322	0.830521	0.846191	0.912491
Mean	0.814984	0.830199	0.84416	0.863167	0.926552
Median	0.8181	0.829819	0.84568	0.861275	0.926353
Standard deviation	0.714943	0.81282	0.710764	0.615804	0.914664

**Table 6:** State-of-the-art analysis of the suggested road accident detection and tracking approach.

TERMS	COA- AYSIDSORT [44]	FFO- AYSIDSORT [45]	OOA- AYSIDSORT [43]	EO- AYSIDSORT [42]	GP-EO- AYSIDSORT
**Dataset 1**					
Best	1.37822	1.272707	1.255195	1.257214	1.014871
Worst	3.485438	3.860697	2.110395	1.786413	1.903607
Mean	1.676592	1.499405	1.347626	1.308274	1.102741
Median	1.596221	1.380251	1.340763	1.270432	1.014871
Standard deviation	0.47081	0.412243	0.120589	0.141111	0.263687
TERMS	**YOLOv8 [49]**	**YOLOv10 [50]**	**Fuzzy vision-based** **DNN [28]**	**AYSIDSORT**	**GP-EO-** **AYSIDSORT**
Best	86.58228	81.51899	87.8481	86.58228	92.40506
Worst	89.11392	83.5443	86.07595	92.40506	96.96203
Mean	75.6962	80.50633	86.58228	86.83544	93.92405
Median	80.25316	82.53165	89.87342	84.3038	91.39241
Standard deviation	78.98734	82.53165	86.07595	90.37975	94.43038

- **Best:** It denotes the highest accuracy attained over recurrent empirical runs.
- **Worst:** It denotes the lower accuracy attained over recurrent empirical runs.
- **Mean:** It is the mean accuracy over all runs.
- **Median:** It is the middle value after categorizing all verified accuracy values.
- **Standard Deviation:** It computes the dissimilarity and consistency of the attained accuracy values.

From the results, the proposed AYSIDSORT model detects the accident from the surveillance video with an accuracy of 0.95, whereas the conventional models provide the accuracy of 00.874621, 0.888725, 0.883353, 0.895835 in the road accident detection, which is comparatively lower than the proposed AYSIDSORT. Thus, the designed AYSIDSORT is used for preventing the tragic loss of human lives by providing the proper responses at the right time. This approach helps to prevent the economic burden worldwide and also reduces the millions of fatalities annually by detecting and tracking accidents on time. Thus, the results indicate that the proposed AYSIDSORT is used for providing valuable insights for the detection and tracking of accidents in the environment.

### 6.9. Evaluation of the deep SORT model using recent metrics

The validation of the Deep SORT model using recent metrics, such as MOTA, IDF1, HOTA, ID switches, FP, and FN, is shown in Table 7. In this research work, to ensure a fair comparison with baseline multi-object tracking approaches, the suggested AYSIDSORT approach is validated to measure identity preservation, tracking, detection accuracy, and stability over video frames. Here, the Multiple Object Tracking Accuracy (MOTA) metric computes the overall tracking accuracy with consideration of identity switches, false negatives, and false positives, and the Identification F1 Score (IDF1) metric validates the stability of object identity protection during the tracking sequence. Accordingly, the Higher Order Tracking Accuracy (HOTA) metrics give a fairness evaluation of detection performance, and ID Switches (IDSW) compute the number of times a tracker mistakenly assigns a new ID to an object. Moreover, the False Positives (FP) present the incorrectly tracked objects that do not exist in the ground truth objects, and the False Negatives (FN) give the lost tracked objects. The proposed AYSIDSORT attains 0.89 MOTA, which shows better vehicle tracking accuracy. Unlike, the baseline SORT, which attains 0.72 MOTA, while IOU Tracker attains 0.70, which limits the tracking ability owing to dependency. Thus, the table results show that the suggested AYSIDSORT approach attains better tracking accuracy and improved identity consistency.

**Table 7:** Validation of the Proposed AYSIDSORT model using recent metrics.

Method	MOTA ↑	IDF1 ↑	HOTA ↑	ID Switches ↓	FP ↓	FN ↓
Dataset 1						
SORT	0.72	0.69	0.61	138	790	860
IOU Tracker	0.7	0.66	0.59	152	820	890
Kalman Filter Tracker	0.74	0.71	0.63	125	760	830
Deep SORT (Baseline)	0.83	0.8	0.72	88	600	610
Proposed (AYSIDSORT)	0.89	0.86	0.79	48	390	450
Dataset 2						
SORT	0.7	0.67	0.59	980	5400	6100
IOU Tracker	0.68	0.65	0.57	1120	5700	6400
Kalman Filter Tracker	0.72	0.69	0.61	910	5100	5900
Deep SORT (Baseline)	0.84	0.81	0.73	640	4200	4300
Proposed (AYSIDSORT)	0.9	0.87	0.8	320	2900	3100

### 6.10. Performance validation of the suggested approach using classification and detection metrics

The performance validation of the suggested approach using classification and detection metrics, such as mAP-based metrics, is shown in Table 8. From Table 8, the Average Precision at IoU 0.5 (AP@0.5) computes the detection accuracy while predictions overlap ground truth by at least 50%. This denotes higher detection quality, and also the Mean Average Precision at IoU 0.5 (mAP@0.5) shows the overall detection performance over all objects at IoU threshold 0.5. Thus, the suggested GP-EO-AYSIDSORT efficiently sustain robust detection performance across IoU thresholds, which is more reliable for real-world surveillance scenarios.

**Table 8:** Performance validation of the suggested approach using classification and detection metrics, such as mAP-based metrics.

Metrics	YOLOv8 [49]	YOLOv10 [50]	Fuzzy vision-based DNN [28]	AYSIDSORT	GP-EO-AYSIDSORT
Dataset 1					
AP@0.5	89.87421	88.02401	84.97653	84.97653	84.97653
mAP@0.5	91.92111	88.97407	88.6518	88.6518	88.6518
mAP@0.75	73.34342	76.54576	72.93848	72.93848	72.93848
mAP@0.5:0.95	51.53609	65.51023	64.95283	64.95283	64.95283
Dataset 2					
AP@0.5	84.13048	88.56242	83.15598	84.40697	83.15598
mAP@0.5	89.60021	89.60908	92.29682	90.46991	92.29682
mAP@0.75	72.27633	76.82146	83.63352	84.52368	83.63352
mAP@0.5:0.95	50.91577	64.44964	53.94517	55.72261	53.94517

### 6.11. Validation of the developed traffic surveillance video analysis using recent techniques

The estimation of the developed traffic surveillance video analysis using recent techniques is shown in Table 9. This validation reports the accuracy improvements of the developed method over YOLOv8, YOLOv9, YOLOv10, and LRD-DETR methods. From the table results, the developed method attains a maximum accuracy of 95.34% at 75% learning percentage on Dataset 2. Thus, this efficient performance of the suggested approach makes it suitable for real-time applications.

**Table 9:** Estimation of the developed traffic surveillance video analysis using recent techniques.

Learning Percentage	YOLOv8 [49]	YOLOv9 [38]	YOLOv10 [50]	LRD-DETR [51]	Suggested GP-EO-AYSIDSORT
**Mean Classifier Comparison of dataset 2 Accuracy**					
**Dataset 1**					
35	83.30322	86.87592	88.13049	90.25238	90.48151
45	84.61212	85.06226	89.4046	87.88025	91.54185
55	83.81775	85.64209	87.68799	89.64691	92.60808
65	83.51685	86.95709	88.54034	90.86517	93.67334
75	84.59259	87.51495	86.88965	88.68988	94.77666
**Dataset 2**					
35	85.03967	85.24353	87.7478	89.32343	89.56805
45	83.59711	85.83313	87.28668	89.17755	90.9811
55	83.16284	86.2439	87.62207	89.27979	92.41994
65	83.39417	88.18573	88.31787	89.5752	93.87573
75	85.20935	86.93634	88.88397	90.01343	95.34748

### 6.12. Estimation of Training Time of the Developed Method

The evaluation of training time for the suggested traffic surveillance video validation is shown in Table 10. The core aim of this validation is to explore the computational efficiency of the proposed GP-EO-AYSIDSORT model compared with the traditional approaches. While considering dataset 2, the simulation findings highlight that the suggested method attains 35.06648 (Mins) for completing the task. Thus, the table results show that the suggested model achieves very efficient performance, which helps to support the claim of real-time suitability.

**Table 10:** Evaluation of training time for the suggested traffic surveillance video validation.

Training Time of Classifier Comparison of Dataset 1					
**Methods**	**YOLOv8 [49]**	**YOLOv10 [50]**	**Fuzzy vision-based DNN [28]**	**AYSIDSORT**	**GP-EO-AYSIDSORT**
Training Time (Mins)	38.64591	37.36276	35.57036	35.23109	35.06648
**Training Time of Classifier Comparison of Dataset 2**					
Training Time (Mins)	44.2851	42.0738	41.84578	40.78077	38.84388

## 7. Conclusion

To improve traffic surveillance video validation, this research work developed an AYSIDSORT framework for road accident detection, classification, and vehicle tracking from surveillance videos. Especially, the suggested approach incorporated an adaptive YOLOv9-based detector, SE-InceptionNet feature learning, and Deep SORT tracking to examine accident-related incidents and track vehicles. The proposed AYSIDSORT model detected the accident from the surveillance video with an accuracy of 0.95, whereas the conventional models provided accuracies of 00.874621, 0.888725, 0.883353, and 0.895835 in road accident detection, which was comparatively lower than the proposed AYSIDSORT. Thus, the results showed that the suggested model achieved satisfactory results in road accident prediction and tracking. In future, the system performance will be improved by tuning the parameters of the proposed AYSIDSORT, which will be used to further reduce the false positives in road accident detection and tracking. Especially, we will focus on real-world deployment and field studies to quantify the impact of the suggested framework on response time, traffic safety, and emergency management effectiveness in the upcoming work. In addition, the IoT-based communication and geolocation pipeline has not been experimentally validated, and it will be considered as future work.
